# Identification of Hasegawa Dementia Scale‐Revised Cutoff Scores Associated With Mini‐Mental State Examination Thresholds for Anti‐Amyloid β Therapies in Patients With Amnesia

**DOI:** 10.1111/psyg.70107

**Published:** 2025-10-26

**Authors:** Kei Yamakami, Tomoyasu Matsubara, Koji Fujita, Kazumi Nakamura, Naoki Kihara, Kazuki Sogawa, Ryohei Nakao, Kenta Hanada, Yuki Yamamoto, Shotaro Haji, Yoichi Otomi, Masafumi Harada, Yuishin Izumi

**Affiliations:** ^1^ Department of Neurology Tokushima University Graduate School of Biomedical Sciences Tokushima Japan; ^2^ Department of Radiology Tokushima University Graduate School of Biomedical Sciences Tokushima Japan

**Keywords:** Alzheimer's disease, amyloid, Hasegawa Dementia Scale‐Revised, mental status and dementia tests, mild cognitive impairment, Mini‐Mental State Examination

## Abstract

**Background:**

This study aimed to evaluate Hasegawa Dementia Scale‐Revised (HDS‐R) cutoff scores that correspond to Mini‐Mental State Examination (MMSE) thresholds for identifying candidates for anti‐amyloid β (Aβ) therapies. Additionally, we conducted exploratory analyses to examine the cognitive subdomains associated with Aβ status.

**Methods:**

This retrospective cross‐sectional study included consecutive patients with amnesia who underwent neuropsychological examinations and Aβ assessment through cerebrospinal fluid analysis or positron emission tomography. Diagnostic accuracy for MMSE thresholds (≥ 20, ≥ 22 and ≥ 24) was assessed, and two HDS‐R cutoffs (high‐sensitivity, high‐specificity) were determined for each threshold. We examined differences in cognitive subdomains between Aβ‐positive and Aβ‐negative patients with MMSE score ≥ 20.

**Results:**

Of 234 patients, 143 (61.1%) were Aβ‐positive. The area under the receiver operating characteristic curve for predicting MMSE score ≥ 20, ≥ 22 and ≥ 24 was 0.92, 0.90 and 0.91, respectively. High‐sensitivity cutoffs, defined as scores that maximised specificity while maintaining sensitivity ≥ 80%, were HDS‐R score ≥ 16, ≥ 17 and ≥ 18. Conversely, high‐specificity cutoffs, defined as scores that maximised sensitivity while maintaining specificity ≥ 80%, were HDS‐R score ≥ 19, ≥ 20 and ≥ 21, respectively. Subdomain analysis of MMSE and HDS‐R showed Aβ‐positive patients had lower scores in delayed recall and higher scores in calculation than Aβ‐negative patients (all *p* < 0.01). In HDS‐R subdomains, visual memory scores were also lower in Aβ‐positive patients than in Aβ‐negative patients (*p* < 0.01).

**Conclusions:**

The identified HDS‐R cutoff scores were associated with MMSE‐defined cognitive thresholds and may serve as a potential reference for identifying patients who could be eligible for anti‐Aβ therapies. The cognitive profile observed in Aβ‐positive patients was characterised by deficits in delayed recall and in visual memory and by relatively preserved calculation ability, suggesting a selective vulnerability pattern in early Alzheimer's disease.

## 1 Introduction

Alzheimer's disease (AD) is a progressive neurodegenerative disorder characterised by the deposition of amyloid β (Aβ) and phosphorylated tau proteins, resulting in neuronal loss and cognitive decline [1]. As the leading cause of late‐onset dementia, AD has a substantial socioeconomic burden, emphasising the importance of early detection and timely intervention [2, 3]. The recent introduction of anti‐Aβ monoclonal antibodies as disease‐modifying therapies has represented an advancement in AD management [4, 5]. The Mini‐Mental State Examination (MMSE) [6], an internationally recognised cognitive screening tool, is used to assess eligibility for these treatments based on designated score ranges (e.g., 22–30 for lecanemab and 20–28 for donanemab).

In Japan, the Hasegawa Dementia Scale‐Revised (HDS‐R) is widely used as a cognitive screening tool [7, 8, 9]. It is a concise 9‐item, 30‐point test that requires approximately 10 min to administer. HDS‐R has also been translated and validated in several Asian countries [10, 11, 12, 13, 14]. However, HDS‐R thresholds are not currently incorporated into screening criteria for anti‐Aβ therapies. Establishing a correspondence between MMSE and HDS‐R scores may help clinicians identify eligible patients earlier and more accurately, reducing missed therapeutic opportunities and avoiding unnecessary specialist referrals.

Moreover, previous studies have suggested that subdomain scores on neuropsychological examinations varied in their ability to predict Aβ deposition depending on the patient background. However, evidence remains limited for patients with mild cognitive impairment (MCI) or mild dementia who are potential candidates for anti‐Aβ therapies [15, 16, 17, 18, 19, 20, 21].

In this context, the present study aimed to identify cutoff scores for HDS‐R that correspond to MMSE thresholds. Additionally, we conducted exploratory analyses to examine the association between subdomains of MMSE and HDS‐R and Aβ status to improve the accuracy of identifying potentially eligible patients for anti‐Aβ therapies.

## 2 Methods

### 2.1 Ethics Considerations

This study was approved by the Ethics Committee of Tokushima University Hospital (approval number: 4687–1). Patients were allowed to opt out of the study, and this information was made available on the Tokushima University Hospital website. This study was conducted in accordance with the principles of the Declaration of Helsinki.

### 2.2 Study Design and Participants

We included consecutive patients who presented with amnesia as the chief complaint at Tokushima University Hospital between March 2015 and May 2025, underwent both MMSE and HDS‐R, and underwent cerebrospinal fluid (CSF) analysis or positron emission tomography (PET) examinations to assess Aβ deposition as part of a comprehensive dementia evaluation. Patients were excluded if amnesia was not their chief complaint or if they had completed only one of the two neuropsychological examinations (MMSE or HDS‐R). Clinical data were collected through a chart review by physicians blinded to the neuropsychological examination results. Both neuropsychological examinations were conducted on the same day for each patient and were consistently performed by a board‐certified speech‐language‐hearing therapist (KN). For patients who underwent multiple evaluations during the study period, only the data obtained at their first assessment were included for analysis. In MMSE, attention was consistently assessed using serial subtractions of 7s rather than spelling words backward.

### 2.3 HDS‐R

HDS‐R assesses cognition through 9 components: age (1 point), orientation in time (4 points), orientation in place (2 points), repeating 3 words (3 points), serial subtractions of 7s (2 points), digits backward (2 points), recalling of 3 words (6 points), recalling of 5 objects (5 points) and generating vegetables (5 points), for a total of 30 points [9]. While HDS‐R contains several items similar to those of MMSE, it differs particularly in that it allocates 6 points to delayed recall with partial credit for cued recognition, limits the calculation task to 2 serial subtractions of 7s, incorporates a visual memory test requiring recall of 5 objects, adds a category fluency component in which participants list vegetable names, and omits any reading or writing tasks.

### 2.4 Aβ Assessment

Aβ deposition was assessed via either CSF analysis or amyloid PET. CSF samples were obtained through lumbar puncture, and the first tube was sent for cell counting and routine biochemical testing. For Aβ analysis, subsequent CSF samples were collected into polypropylene tubes and stored at ≤ −20°C. CSF Aβ analysis was performed using either chemiluminescence enzyme immunoassay for the Aβ42/40 ratio (conducted using the LUMIPULSE G β‐Amyloid 1–42 and 1–40 assay kits [Cat#230336/231524] and the LUMIPULSE G1200 plus analyser at Fujirebio Inc., Tokyo, Japan) or enzyme‐linked immunosorbent assay for Aβ42 measurement (conducted using the human/rat β‐Amyloid (1–42) ELISA Kit, High Sensitivity [Cat#290–62 601, FUJIFILM Wako Pure Chemical Corporation, Osaka, Japan] and an Infinite F50R microplate reader [Tecan Group Ltd., Männedorf, Switzerland] at Tokiwa Chemical Industries Co. Ltd., Tokyo, Japan). The cutoff values for Aβ positivity were set at 0.067 for the Aβ42/40 ratio and 500 pg/mL for Aβ42 concentration.

Amyloid PET was performed using either ^18^F‐flutemetamol or ^18^F‐florbetapir. For ^18^F‐flutemetamol, 185 MBq was intravenously administered, and scanning was performed 90 min post‐injection for 30 min. For ^18^F‐florbetapir, 370 MBq was administered with imaging at 50 min post‐injection for 20 min. All scans were acquired on a Discovery 710 PET/CT scanner (GE Healthcare, Chicago, IL, USA) using CT‐based attenuation correction and standard manufacturer‐recommended reconstruction protocols. All PET images were visually assessed for Aβ positivity by two board‐certified nuclear medicine physicians who were blinded to clinical information.

### 2.5 Statistical Analysis

Differences between the groups were analysed using the Mann–Whitney U test for continuous variables and the *χ* ^2^ test for categorical variables. Correlations between continuous variables were evaluated using the Spearman rank correlation coefficient. Covariates identified as significant in univariate analysis were further assessed using multivariate analysis. Sensitivity and specificity were estimated using the Clopper–Pearson exact method with 95% confidence intervals (CIs), assuming a binomial distribution. To evaluate the ability of HDS‐R to predict MMSE thresholds (≥ 20, ≥ 22 and ≥ 24), we performed receiver operating characteristic (ROC) curve analysis and measured the area under the ROC curve (AUC) with 95% CI. These thresholds were selected because the lower cutoff scores in the current screening criteria for anti‐amyloid β therapies are 20 for donanemab and 22 for lecanemab, and 24 has traditionally been used as the cutoff score for MCI. The high‐sensitivity cutoff score was defined as the score that maximised specificity while ensuring that the lower bound of the 95% CI for sensitivity was ≥ 80%; conversely, the high‐specificity cutoff score was defined as the score that maximised sensitivity while ensuring that the lower bound of the 95% CI for specificity was ≥ 80%. To examine the association between MMSE and HDS‐R scores and cognitive subdomains and Aβ status, rank transformation analysis of covariance (ANCOVA) was performed. MMSE and HDS‐R subdomain scores were rank‐transformed before ANCOVA to address potential non‐normal distributions. These subdomain analyses were restricted to patients with MMSE score ≥ 20, corresponding to the lower bound of current MMSE‐based screening criteria for anti‐Aβ therapies and to reduce floor‐effect bias at very low MMSE scores. All statistical tests were two‐sided, with a significance level of 0.05. Results were expressed as mean ± standard deviation (SD) unless specified otherwise. All statistical analyses were conducted using Stata BE 18 (StataCorp LLC, College Station, TX, USA).

## 3 Results

### 3.1 Baseline Characteristics

A total of 234 patients were included in this study. Aβ status was determined using amyloid PET in 137 patients (58.5%), CSF Aβ42/40 ratio in 36 patients (15.4%), and CSF Aβ42 concentration in 61 patients (26.1%). Among them, 143 (61.1%) were Aβ‐positive, and 91 (38.9%) were Aβ‐negative. The baseline clinical characteristics of the study population are summarised in Table 1.

**TABLE 1:** Baseline clinical characteristics of the patients.

	Total (*n* = 234)	Aβ positive (*n* = 143)	Aβ negative (*n* = 91)	*p*	MMSE ≥ 20, total (*n* = 173)	MMSE ≥ 20, Aβ positive (*n* = 97)	MMSE ≥ 20, Aβ negative (*n* = 76)	*p*
Age at exam, mean ± SD, years	73.4 ± 9.2	73.1 ± 8.8	73.8 ± 9.9	0.34	73.4 ± 9.4	73.5 ± 9.0	73.4 ± 10.1	0.77
Sex (M: F)	101:133	60:83	41:50	0.64	79:94	43:54	36:40	0.69
Education, mean ± SD, years	12.7 ± 2.4^a^	12.9 ± 2.4^b^	12.5 ± 2.4^c^	0.18	12.8 ± 2.4	13.1 ± 2.4	12.4 ± 2.4	0.07
Interval between onset and exam, median [IQR], years	2.1 [3.4]	2.1 [3.5]	2.1 [3.8]	0.71	1.8 [3.0]	1.8 [2.3]	2.0 [3.6]	0.69
MMSE, mean ± SD	22.1 ± 4.2	21.2 ± 3.8	23.7 ± 4.3	< 0.001	24.0 ± 2.8	23.2 ± 2.4	25.1 ± 2.9	< 0.001
HDS‐R, mean ± SD	18.9 ± 5.3	17.4 ± 4.5	21.3 ± 5.6	< 0.001	20.9 ± 4.4	19.4 ± 3.5	22.8 ± 4.6	< 0.001
Use of anti‐dementia drugs before exam	82	62	20	0.001	46	33	13	0.01
Hypertension	115	74	41	0.32	92	52	40	0.90
Diabetes	26	14	12	0.42	21	11	10	0.72

*Note:* Continuous variables are presented as mean ± SD unless otherwise specified. Interval between onset and exam, which showed a particularly skewed distribution (Figure S2), is presented as median [IQR].

Abbreviations: Aβ, amyloid β; F, female; HDS‐R, Hasegawa Dementia Scale‐Revised; IQR, interquartile range; M, male; MMSE, Mini‐Mental State Examination; SD, standard deviation.

^a^ *n* = 215.

^b^ *n* = 131.

^c^ *n* = 84.

### 3.2 Diagnostic Performance of HDS‐R for Predicting MMSE Status

**FIGURE 1:**
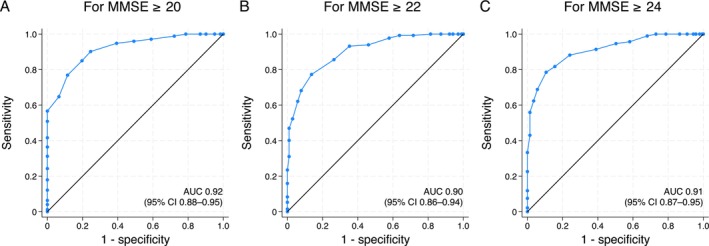
ROC curves for predicting MMSE thresholds using HDS‐R scores. ROC curves for predicting MMSE score ≥ 20 (A), ≥ 22 (B) and ≥ 24 (C) based on HDS‐R scores. The AUC values are 0.92 (95% CI: 0.88–0.95), 0.90 (95% CI: 0.86–0.94) and 0.91 (95% CI: 0.87–0.95), respectively. AUC, area under the receiver operating characteristic curve; CI, confidence interval; HDS‐R, Hasegawa Dementia Scale‐Revised; MMSE, Mini‐Mental State Examination; ROC, receiver operating characteristic.

As context for the relationship between the two scales, a scatter plot for the entire cohort is provided in Figure S1. The diagnostic performance of HDS‐R in predicting MMSE scores of ≥ 20, ≥ 22 and ≥ 24, as determined by ROC curve analysis, is shown in Figure 1. The AUC values were 0.92 (95% CI: 0.88 to 0.95) for MMSE score ≥ 20, 0.90 (95% CI: 0.86 to 0.94) for MMSE score ≥ 22, and 0.91 (95% CI: 0.87 to 0.95) for MMSE score ≥ 24.

We evaluated the diagnostic performance of HDS‐R in predicting different MMSE thresholds (MMSE score ≥ 20, ≥ 22 and ≥ 24) and summarised the results in Table 2. Sensitivity and specificity were calculated for each cutoff of HDS‐R score (Table S1).

**TABLE 2:** Diagnostic performance of HDS‐R in predicting different MMSE thresholds.

MMSE threshold	Operating point	HDS‐R cutoff	Sn (95% CI)	Sp (95% CI)	PPV (95% CI)	NPV (95% CI)	LR+ (95% CI)	LR− (95% CI)
For MMSE ≥ 20	High sensitivity	15/16	90.2 (84.7, 94.2)	75.4 (62.7, 85.5)	91.2 (85.9, 95.0)	73.0 (60.3, 83.4)	3.7 (2.4, 5.7)	0.13 (0.08, 0.21)
	High specificity	18/19	64.7 (57.1, 71.8)	93.4 (84.1, 98.2)	96.6 (91.4, 99.1)	48.3 (39.0, 57.7)	9.9 (3.8, 25.6)	0.38 (0.31, 0.47)
For MMSE ≥ 22	High sensitivity	16/17	93.2 (87.5, 96.8)	64.7 (54.6, 73.9)	77.4 (70.1, 83.6)	88.0 (78.4, 94.4)	2.6 (2.0, 3.5)	0.11 (0.06, 0.20)
	High specificity	19/20	68.2 (59.5, 76.0)	92.2 (85.1, 96.6)	91.8 (84.5, 96.4)	69.1 (60.6, 76.8)	8.7 (4.4, 17.1)	0.35 (0.27, 0.45)
For MMSE ≥ 24	High sensitivity	17/18	91.4 (83.8, 96.2)	61.0 (52.4, 69.1)	60.7 (52.1, 68.9)	91.5 (83.9, 96.3)	2.3 (1.9, 2.9)	0.14 (0.07, 0.28)
	High specificity	20/21	78.5 (68.8, 86.3)	89.4 (83.1, 93.9)	83.0 (73.4, 90.1)	86.3 (79.6, 91.4)	7.4 (4.5, 12.0)	0.24 (0.16, 0.36)

*Note:* High sensitivity cutoff: the cutoff that maximised specificity while ensuring that the lower bound of the 95% CI for sensitivity was at least 80%. High specificity cutoff: the cutoff that maximised sensitivity while ensuring that the lower bound of the 95% CI for specificity was at least 80%.

Abbreviations: CI, confidence interval; HDS‐R, Hasegawa Dementia Scale‐Revised, LR+, positive likelihood ratio; LR**−**, negative likelihood ratio; MMSE, Mini‐Mental State Examination; NPV, negative predictive value; PPV, positive predictive value; Sn, sensitivity; Sp, specificity.

For MMSE score ≥ 20, the HDS‐R high‐sensitivity cutoff score was ≥ 16, with a sensitivity of 90.2% (95% CI: 84.7% to 94.2%) and a specificity of 75.4% (95% CI: 62.7% to 85.5%). The HDS‐R high‐specificity cutoff score was ≥ 19, yielding a sensitivity of 64.7% (95% CI: 57.1% to 71.8%) and a specificity of 93.4% (95% CI: 84.1% to 98.2%).

For MMSE score ≥ 22, the HDS‐R high‐sensitivity cutoff score was ≥ 17, with a sensitivity of 93.2% (95% CI: 87.5% to 96.8%) and a specificity of 64.7% (95% CI: 54.6% to 73.9%). The HDS‐R high‐specificity cutoff score was ≥ 20, with a sensitivity of 68.2% (95% CI: 59.5% to 76.0%) and a specificity of 92.2% (95% CI: 85.1% to 96.6%).

For MMSE score ≥ 24, the HDS‐R high‐sensitivity cutoff score was ≥ 18, yielding a sensitivity of 91.4% (95% CI: 83.8% to 96.2%) and a specificity of 61.0% (95% CI: 52.4% to 69.1%). The HDS‐R high‐specificity cutoff score was ≥ 21, yielding a sensitivity of 78.5% (95% CI: 68.8% to 86.3%) and a specificity of 89.4% (95% CI: 83.1% to 93.9%).

### 3.3 Association Between Cognitive Subdomains and Aβ Status

Among the 173 patients included, the mean MMSE score was 24.0 ± 2.8, and 93 patients (54%) had scores within the conventional MCI range (MMSE score ≥ 24). On the basis of the results summarised in Table 1 and previous reports [22, 23, 24], we selected the covariates for adjustment. MMSE score, dementia medication use, age and years of education were included as covariates, as MMSE score and dementia medication use showed significant differences between the Aβ‐positive and Aβ‐negative groups, and age and years of education have been reported to influence cognitive performance. Although HDS‐R scores also differed significantly between the groups, they were strongly correlated with MMSE scores (Spearman *ρ* = 0.75), potentially leading to concerns about multicollinearity if both were included. Furthermore, HDS‐R scores were more strongly correlated with Aβ status (Spearman *ρ* = −0.39) than MMSE scores (Spearman *ρ* = −0.32), suggesting that adjusting for HDS‐R scores could lead to overadjustment, potentially obscuring the association between Aβ status and specific cognitive subdomains. Therefore, we included MMSE score as a covariate but excluded HDS‐R score to minimise the risk of overadjustment.

Among MMSE subdomains, calculation ability was relatively preserved in the Aβ‐positive group compared with the Aβ‐negative group (estimate: 20.9, 95% CI: 7.6 to 34.2, *p* = 0.002). In contrast, delayed recall was significantly lower in the Aβ‐positive group than in the Aβ‐negative group (estimate: −16.7, 95% CI: −27.7 to −5.8, *p* = 0.003). No significant differences were observed in the other subdomains (Table 3).

**TABLE 3:** Analysis of MMSE cognitive subdomains by amyloid β status with covariate adjustment.

Subdomain	Estimate (95% CI)	*p*
Orientation (time)	−7.9 (−20.1, 4.4)	0.21
Orientation (place)	−11.5 (−24.0, 0.9)	0.07
Immediate recall (repeating 3 words)	−0.1 (−5.4, 5.2)	0.96
Calculation (serial subtractions of 7s)	20.9 (7.6, 34.2)	0.002
Delayed recall (recalling of 3 words)	−16.7 (−27.7, −5.8)	0.003
Naming	ND	
Repetition	7.6 (−0.8, 15.9)	0.08
Three‐step command	3.5 (−9.7, 16.6)	0.60
Reading	ND	
Writing	6.5 (−0.4, 13.4)	0.07
Visuospatial ability	−6.9 (−17.3, 3.5)	0.19

Abbreviations: CI, confidence interval; MMSE, Mini‐Mental State Examination; ND, no difference.

Regarding HDS‐R subdomains, calculation ability was relatively preserved in the Aβ‐positive group compared with the Aβ‐negative group (estimate: 17.3, 95% CI: 6.0 to 28.6, *p* = 0.003), as in MMSE subdomains. Delayed recall was also significantly lower in the Aβ‐positive group than in the Aβ‐negative group (estimate: −20.8, 95% CI: −32.4 to −9.3, *p* < 0.001). Furthermore, visual memory was significantly lower in the Aβ‐positive group than in the Aβ‐negative group (estimate: −19.2, 95% CI: −33.3 to −5.1, *p* = 0.008). No significant differences were observed in the other subdomains (Table 4).

**TABLE 4:** Analysis of HDS‐R cognitive subdomains by amyloid β status with covariate adjustment.

Subdomain	Estimate (95% CI)	*p*
Age	−1.2 (−6.0, 3.7)	0.64
Orientation (time)	−7.4 (−19.8, 5.1)	0.25
Orientation (place)	3.2 (−4.2, 10.6)	0.40
Immediate recall (repeating 3 words)	−0.1 (−5.4, 5.2)	0.96
Calculation (serial subtractions of 7s)	17.3 (6.0, 28.6)	0.003
Working memory (digits backward)	6.6 (−8.1, 21.3)	0.38
Delayed recall (recalling of 3 words)	−20.8 (−32.4, −9.3)	< 0.001
Visual memory (recalling of 5 objects)	−19.2 (−33.3, −5.1)	0.008
Category fluency (generating vegetables)	−6.5 (−21.1, 8.0)	0.38

Abbreviations: CI, confidence interval; HDS‐R, Hasegawa Dementia Scale‐Revised.

## 4 Discussion

This study identified cutoff scores for HDS‐R that can predict MMSE thresholds with high accuracy, providing a potential reference for identifying patients who may be eligible for anti‐Aβ therapies. Exploratory subdomain analysis revealed a distinct cognitive profile in Aβ‐positive patients: delayed recall and visual memory were significantly impaired, whereas calculation ability was relatively preserved. This contrast highlights a characteristic cognitive pattern in the early stage of AD, which may aid in screening candidates for anti‐Aβ therapies.

The present study aimed to predict whether a patient's cognitive condition meets the target MMSE range rather than estimate an equivalent MMSE score from a specific HDS‐R score. We chose this approach because anti‐Aβ therapies have drug‐specific eligibility cutoff scores. For instance, lecanemab is approved for patients with an MMSE score ≥ 22, and donanemab is approved for those with scores ranging 20 to 28. Given these eligibility criteria, accurately identifying patients who meet the thresholds before biomarker testing or treatment is crucial for efficient patient selection. In early validation studies after its development, HDS‐R showed a sensitivity of 90% and a specificity of 82% for detecting dementia when a cutoff of 20/21 was applied [9]. Similarly, MMSE showed a sensitivity of 87% and a specificity of 82% when a cutoff of 23/24 was applied [25]. A subsequent study comparing MMSE and HDS‐R within the same cohort showed that an HDS‐R cutoff of 20 had a sensitivity of 67% and a specificity of 89% for dementia detection, while an MMSE cutoff of 23 had a sensitivity of 64% and a specificity of 87% [26]. These findings suggest that an HDS‐R score of 20 corresponds approximately to an MMSE score of 23. However, data have been lacking regarding whether a similar correspondence remains valid across higher or lower score ranges. In fact, the correlations between MMSE and HDS‐R scores vary across studies, with some reporting a strong correlation (coefficient = 0.94) [9] and others indicating a moderate correlation (coefficient = 0.435) [22]; our cohort showed a correlation coefficient of 0.75. This interstudy variability raises concerns about the reliability of using regression for direct score‐to‐score conversion. In contrast, predicting whether a score meets a defined threshold (a classification task) is a more robust approach, as it is less susceptible to interstudy variability. Therefore, this study's method of classifying patients on the basis of eligibility criteria may offer a more practical and reliable framework than attempting to establish a precise score equivalence.

**FIGURE 2:**
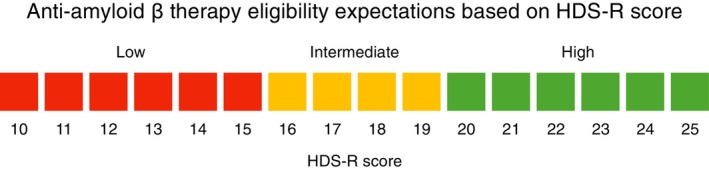
A framework for stratifying anti‐amyloid β therapy eligibility based on HDS‐R score. The figure illustrates a data‐driven framework for stratifying patients into 3 probability groups (Low, Intermediate and High) for potential MMSE‐based eligibility. The classification is based on two optimised HDS‐R cutoff scores derived from Table 2. The ‘Low probability’ group is defined by scores below a high‐sensitivity cutoff score (maximising specificity while ensuring the lower bound of the 95% CI for sensitivity ≥ 80%). The ‘High probability’ group is defined by scores at or above a high‐specificity cutoff score (maximising sensitivity while ensuring the lower bound of the 95% CI for specificity ≥ 80%). This approach provides a standardised method to support clinical screening for anti‐Aβ therapies. The Low, Intermediate and High groups in this schema correspond to approximate MMSE ranges. Based on the operating points in Table 2, patients in the High group are likely to have MMSE score ≥ 22 (specificity 92.2%), whereas those in the Low group are likely to have MMSE score < 20 (sensitivity for MMSE score ≥ 20: 90.2%). These ranges are approximate and not intended for exact score conversion. HDS‐R, Hasegawa Dementia Scale‐Revised; MMSE, Mini‐Mental State Examination.

HDS‐R cutoff scores identified in this study can offer a framework for stratifying patients into probability groups for anti‐Aβ therapy eligibility, as illustrated in Figure 2. Specifically, by utilising the optimal cutoffs presented in Table 2, a two‐tiered threshold system can be established. Scores below the high‐sensitivity cutoff (e.g., HDS‐R score < 16 for predicting MMSE score ≥ 20) would classify patients into a ‘Low probability’ group, suggesting they are unlikely to meet the eligibility criteria. Conversely, scores at or above the high‐specificity cutoff (e.g., HDS‐R score ≥ 19 for predicting MMSE score ≥ 22) would identify a ‘High probability’ group. Patients with scores falling between these two thresholds would be categorised as ‘Intermediate probability’. This stratification, which integrates both high‐sensitivity and high‐specificity cutoffs, provides a balanced approach to minimising false negatives and false positives. In countries such as Japan, where HDS‐R is a widely used cognitive screening tool, such a system could streamline the identification of potentially eligible patients for anti‐Aβ therapies, inform referral strategies and help optimise the diagnostic workflow in clinical practice.

In both MMSE and HDS‐R, scores in delayed recall were consistently lower and scores in calculation were consistently higher in the Aβ‐positive group than in the Aβ‐negative group. Although the relative contributions of these subdomains to the total score differ between MMSE and HDS‐R (HDS‐R allocates 6 points to delayed recall compared with 3 in MMSE, and 2 points to calculation compared with 5 in MMSE), significant differences have been observed beyond the structural differences between these test batteries. Previous studies showed that patients with AD had significantly worse scores on the memory subdomain but better scores on the attention subdomain [27, 28]. These findings are consistent with ours. However, in contrast to previous studies that identified deficits in construction ability (assessed as visuospatial ability in MMSE) [28], this study found no significant difference between the Aβ‐positive and Aβ‐negative groups. One possible explanation is that previous studies primarily included patients with mild dementia, whereas our cohort included a substantial proportion of patients with MCI‐equivalent scores. Given that tau accumulation in AD begins in the temporal lobe and subsequently spreads to extratemporal cortical regions [29, 30], deficits in calculation and construction, functions that are also associated with the parietal lobe, are expected to emerge later than deficits in delayed recall. Additionally, HDS‐R includes a unique visual memory subdomain (5 points) that has no equivalent in MMSE, and this subdomain showed a significant deficit in Aβ‐positive patients. Although this task is visuoperceptual in nature, it also reflects memory and recall. The observed deficit may be attributed to the cognitive load imposed by recalling 5 items rather than a specific visuoperceptual deficit. However, further validation is necessary to confirm this interpretation.

This study has some limitations. First, the study population included only patients who presented with amnesia as their primary symptom. Although amnesia is the most common initial manifestation of AD [31], it remains uncertain whether these findings can be applied to patients with non‐amnestic presentations, such as those with primary language or visuospatial deficits. Second, this study was conducted at a single centre and used a retrospective design. It was also conducted in a Japanese population, and the generalisability of our findings to other ethnic groups has not been validated. Thus, further validation in more diverse, independent cohorts with prospective designs is needed to confirm the present findings, including other ethnic groups and patients with non‐amnestic clinical presentations. Third, the diagnostic modalities used to assess Aβ deposition were not uniform among participants, which might introduce some heterogeneity. Although all of these modalities, including amyloid PET, CSF Aβ42/40 ratio and CSF Aβ42, are widely accepted for determining Aβ positivity [32, 33], future studies are needed to determine whether such differences influence the validity of the findings of this study.

In conclusion, this study identified HDS‐R cutoff scores that can predict MMSE thresholds, providing a reference for identifying patients who may be eligible for anti‐Aβ therapies. Furthermore, the cognitive profile observed in Aβ‐positive patients, including deficits in delayed recall and in visual memory and by relatively preserved calculation ability, suggests a characteristic pattern of cognitive changes in early AD. Recognising these domain‐specific deficits may enhance screening strategies and support earlier detection of AD‐related conditions.

## Consent

Due to the retrospective nature of the study and the use of anonymised data, the requirement for individual informed consent was waived by the ethics committee. Information regarding the study and the opportunity to opt out was provided via the institutional website.

## Conflicts of Interest

Yuishin Izumi has received lecture fees from Eisai Co. Ltd. and Eli Lilly Japan K.K. The other authors declare no conflicts of interest.

## Supporting information


**Figure S1:** Scatter (bubble) plot of Mini‐Mental State Examination (MMSE) scores against Hasegawa Dementia Scale‐Revised (HDS‐R) scores. Each bubble represents one observed score pair; the bubble area is proportional to the number of identical score pairs.

**Figure S2:** Kernel density plots of the interval between symptom onset and neuropsychological examinations. The distribution is shown for the total cohort (*n* = 234) (A) and for the subgroup with MMSE ≥ 20 (*n* = 173) (B), stratified by amyloid status. The vertical axis represents patient probability density estimated by kernel density. MMSE, Mini‐Mental State Examination.

**Table S1:** HDS‐R sensitivity and specificity for MMSE scores 20, 22 and 24.

## Data Availability

The data that support the findings of this study are available from the corresponding author upon reasonable request.
