# Supplementary figures and images for: Identification of Hasegawa Dementia Scale‐Revised Cutoff Scores Associated With Mini‐Mental State Examination Thresholds for Anti‐Amyloid β Therapies in Patients With Amnesia
Source: Psychogeriatrics. 2025 Oct 26;25(6):e70107. doi: 10.1111/psyg.70107 (PMC12555022; doi:10.1111/psyg.70107)

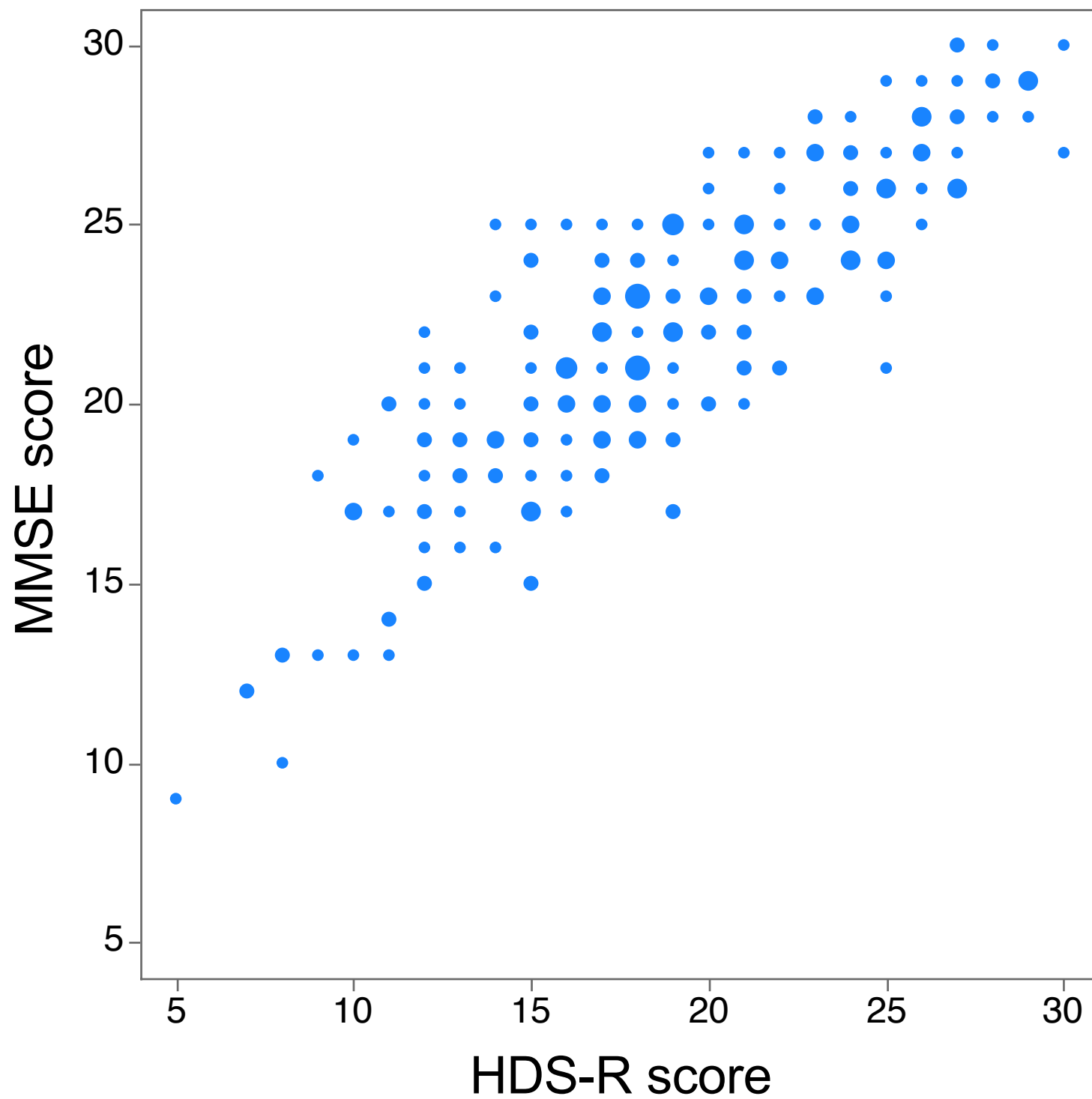

Supplement: Supplementary file 1 — Figure S1: Scatter (bubble) plot of Mini‐Mental State Examination (MMSE) scores against Hasegawa Dementia Scale‐Revised (HDS‐R) scores. Each bubble represents one observed score pair; the bubble area is proportional to the number of identical score pairs. [file PSYG-25-0-s002.pdf]

**A**

Total (n = 234)

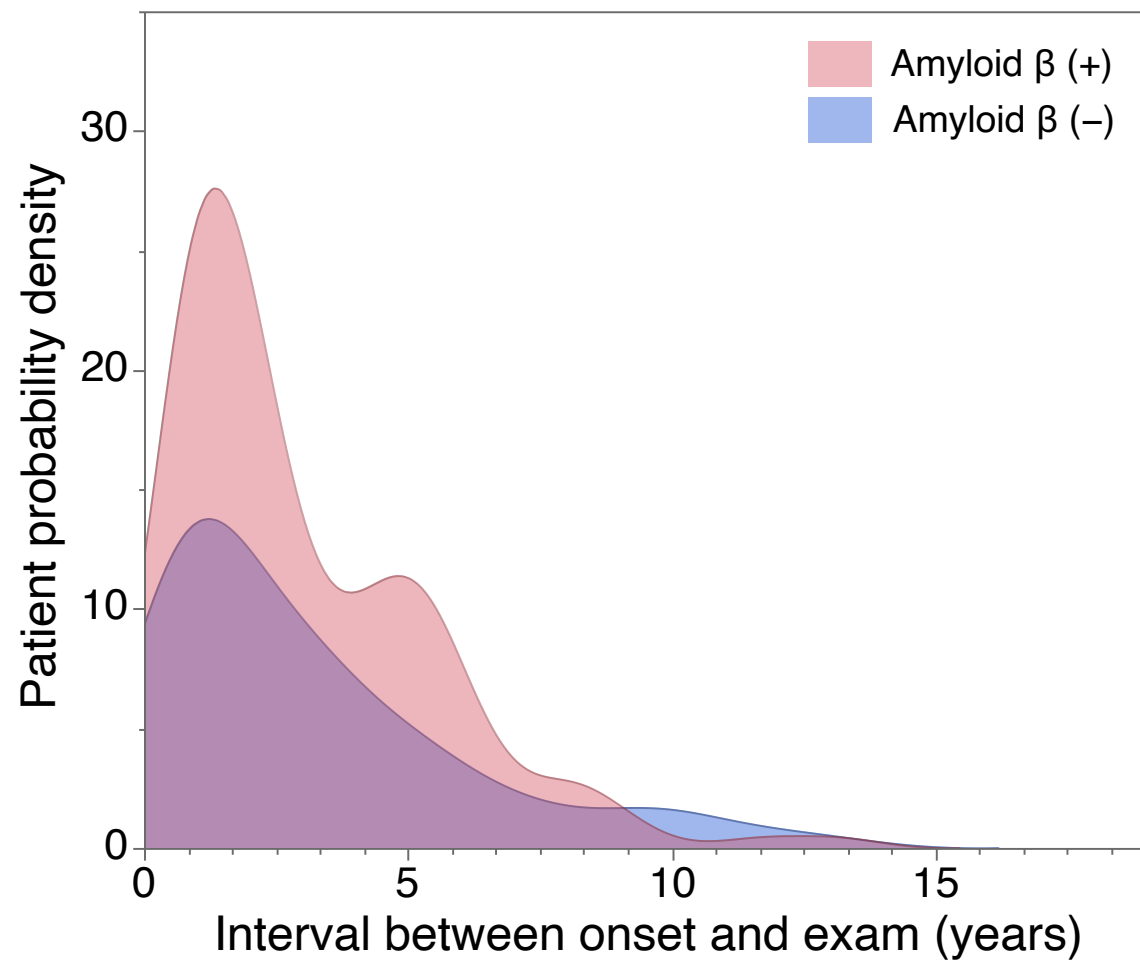**B**MMSE  $\geq 20$  (n = 173)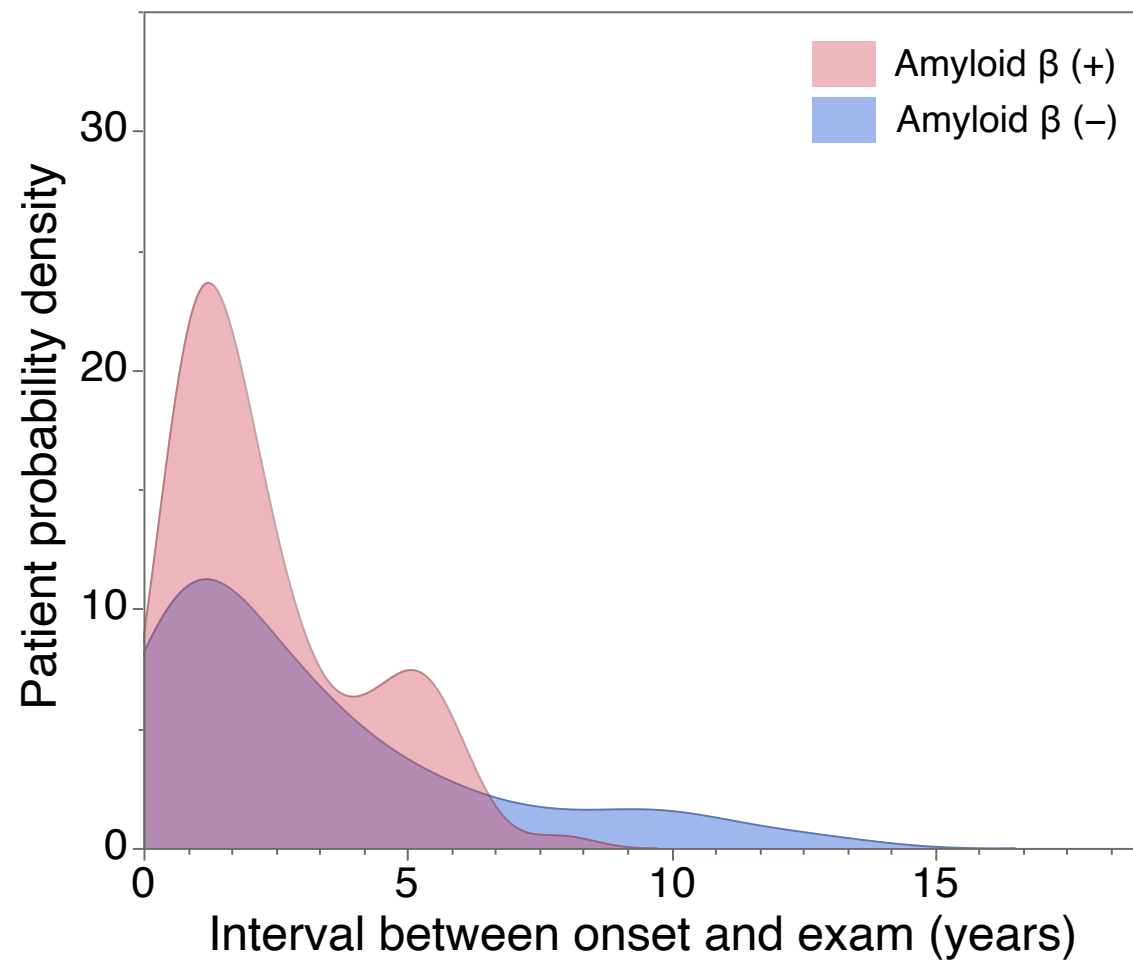

Supplement: Supplementary file 2 — Figure S2: Kernel density plots of the interval between symptom onset and neuropsychological examinations. The distribution is shown for the total cohort (n = 234) (A) and for the subgroup with MMSE ≥ 20 (n = 173) (B), stratified by amyloid status. The vertical axis represents patient probability density estimated by kernel density. MMSE, Mini‐Mental State Examination. [file PSYG-25-0-s003.pdf]
