# Supplementary material for: Identification of Hasegawa Dementia Scale‐Revised Cutoff Scores Associated With Mini‐Mental State Examination Thresholds for Anti‐Amyloid β Therapies in Patients With Amnesia
Source: Psychogeriatrics. 2025 Oct 26;25(6):e70107. doi: 10.1111/psyg.70107 (PMC12555022; doi:10.1111/psyg.70107)
Supplement: Supplementary file 3 — Table S1: HDS‐R sensitivity and specificity for MMSE scores 20, 22 and 24. [file PSYG-25-0-s001.docx]

**Supplementary Table 1: HDS-R sensitivity and specificity for MMSE scores 20, 22, and 24**

| **HDS-R**  **cutoff score for MMSE**  **≥20** | **Sn (95% CI), %** | **Sp (95% CI), %** | **HDS-R**  **cutoff score for MMSE**  **≥22** | **Sn (95% CI), %** | **Sp (95% CI), %** | **HDS-R**  **cutoff score for MMSE**  **≥24** | **Sn (95% CI), %** | **Sp (95% CI), %** |
| --- | --- | --- | --- | --- | --- | --- | --- | --- |
| 9/10 | 100.0 (97.9, 100.0) | 13.1 (5.8, 24.2) | 9/10 | 100.0 (97.2, 100.0) | 7.8 (3.4, 14.9) | 9/10 | 100.0 (96.1, 100.0) | 5.7 (2.5, 10.9) |
| 10/11 | 100.0 (97.9, 100.0) | 21.3 (11.9, 33.7) | 10/11 | 100.0 (97.2, 100.0) | 12.7 (7.0, 20.8) | 10/11 | 100.0 (96.1, 100.0) | 9.2 (5.0, 15.3) |
| 11/12 | 98.8 (95.9, 99.9) | 27.9 (17.1, 40.8) | 11/12 | 100.0 (97.2, 100.0) | 18.6 (11.6, 27.6) | 11/12 | 100.0 (96.1, 100.0) | 13.5 (8.3, 20.2) |
| 12/13 | 97.1 (93.4, 99.1) | 41.0 (28.6, 54.3) | 12/13 | 99.2 (95.9, 100.0) | 28.4 (19.9, 38.2) | 12/13 | 100.0 (96.1, 100.0) | 21.3 (14.8, 29.0) |
| 13/14 | 96.0 (91.8, 98.4) | 50.8 (37.7, 63.9) | 13/14 | 99.2 (95.9, 100.0) | 36.3 (27.0, 46.4) | 13/14 | 100.0 (96.1, 100.0) | 27.0 (19.8, 35.1) |
| 14/15 | 94.8 (90.4, 97.6) | 60.7 (47.3, 72.9) | 14/15 | 97.7 (93.5, 99.5) | 42.2 (32.4, 52.3) | 14/15 | 98.9 (94.2, 100.0) | 31.9 (24.3, 40.3) |
| 15/16† | 90.2 (84.7, 94.2) | 75.4 (62.7, 85.5) | 15/16 | 93.9 (88.4, 97.3) | 53.9 (43.8, 63.8) | 15/16 | 95.7 (89.4, 98.8) | 41.8 (33.6, 50.4) |
| 16/17 | 85.0 (78.8, 89.9) | 80.3 (68.2, 89.4) | 16/17† | 93.2 (87.5, 96.8) | 64.7 (54.6, 73.9) | 16/17 | 94.6 (87.9, 98.2) | 49.6 (41.1, 58.2) |
| 17/18 | 76.9 (69.9, 82.9) | 88.5 (77.8, 95.3) | 17/18 | 85.6 (78.4, 91.1) | 73.5 (63.9, 81.8) | 17/18† | 91.4 (83.8, 96.2) | 61.0 (52.4, 69.1) |
| 18/19‡ | 64.7 (57.1, 71.8) | 93.4 (84.1, 98.2) | 18/19 | 77.3 (69.2, 84.1) | 86.3 (78.0, 92.3) | 18/19 | 88.2 (79.8, 93.9) | 75.9 (68.0, 82.7) |
| 19/20 | 56.6 (48.9, 64.1) | 100.0 (94.1, 100.0) | 19/20‡ | 68.2 (59.5, 76.0) | 92.2 (85.1, 96.6) | 19/20 | 81.7 (72.4, 89.0) | 84.4 (77.3, 90.0) |
| 20/21 | 50.9 (43.2, 58.5) | 100.0 (94.1, 100.0) | 20/21 | 62.1 (53.3, 70.4) | 94.1 (87.6, 97.8) | 20/21‡ | 78.5 (68.8, 86.3) | 89.4 (83.1, 93.9) |
| 21/22 | 41.6 (34.2, 49.3) | 100.0 (94.1, 100.0) | 21/22 | 52.3 (43.4, 61.0) | 97.1 (91.6, 99.4) | 21/22 | 68.8 (58.4, 78.0) | 94.3 (89.1, 97.5) |
| 22/23 | 36.4 (29.2, 44.1) | 100.0 (94.1, 100.0) | 22/23 | 47.0 (38.2, 55.8) | 99.0 (94.7, 100.0) | 22/23 | 62.4 (51.7, 72.2) | 96.5 (91.9, 98.8) |
| 23/24 | 31.2 (24.4, 38.7) | 100.0 (94.1, 100.0) | 23/24 | 40.2 (31.7, 49.0) | 99.0 (94.7, 100.0) | 23/24 | 55.9 (45.2, 66.2) | 98.6 (95.0, 99.8) |
| 24/25 | 24.3 (18.1, 31.4) | 100.0 (94.1, 100.0) | 24/25 | 31.1 (23.3, 39.7) | 99.0 (94.7, 100.0) | 24/25 | 43.0 (32.8, 53.7) | 98.6 (95.0, 99.8) |
| 25/26 | 17.9 (12.5, 24.5) | 100.0 (94.1, 100.0) | 25/26 | 23.5 (16.5, 31.6) | 100.0 (96.4, 100.0) | 25/26 | 33.3 (23.9, 43.9) | 100.0 (97.4, 100.0) |
| 26/27 | 12.1 (7.7, 18.0) | 100.0 (94.1, 100.0) | 26/27 | 15.9 (10.1, 23.3) | 100.0 (96.4, 100.0) | 26/27 | 22.6 (14.6, 32.4) | 100.0 (97.4, 100.0) |
| 27/28 | 6.4 (3.2, 11.1) | 100.0 (94.1, 100.0) | 27/28 | 8.3 (4.2, 14.4) | 100.0 (96.4, 100.0) | 27/28 | 11.8 (6.1, 20.2) | 100.0 (97.4, 100.0) |
| 28/29 | 4.0 (1.6, 8.2) | 100.0 (94.1, 100.0) | 28/29 | 5.3 (2.2, 10.6) | 100.0 (96.4, 100.0) | 28/29 | 7.5 (3.1, 14.9) | 100.0 (97.4, 100.0) |
| 29/30 | 1.2 (0.1, 4.1) | 100.0 (94.1, 100.0) | 29/30 | 1.5 (0.2, 5.4) | 100.0 (96.4, 100.0) | 29/30 | 2.2 (0.3, 7.6) | 100.0 (97.4, 100.0) |

†The cutoff that maximised specificity while ensuring that the lower bound of the 95% CI for sensitivity was at least 80%.

‡The cutoff that maximised sensitivity while ensuring that the lower bound of the 95% CI for specificity was at least 80%.

CI, confidence interval; Sn, sensitivity; Sp, specificity; MMSE, Mini-Mental State Examination; HDS-R, Hasegawa Dementia Scale-Revised
